# From perception to behavior: The neural circuits underlying prey hunting in larval zebrafish

**DOI:** 10.3389/fncir.2023.1087993

**Published:** 2023-02-01

**Authors:** Shuyu I. Zhu, Geoffrey J. Goodhill

**Affiliations:** Departments of Developmental Biology and Neuroscience, Washington University in St. Louis, St. Louis, MO, United States

**Keywords:** optic tectum, vision, neural coding, internal states, experience-dependent plasticity

## Abstract

A key challenge for neural systems is to extract relevant information from the environment and make appropriate behavioral responses. The larval zebrafish offers an exciting opportunity for studying these sensing processes and sensory-motor transformations. Prey hunting is an instinctual behavior of zebrafish that requires the brain to extract and combine different attributes of the sensory input and form appropriate motor outputs. Due to its small size and transparency the larval zebrafish brain allows optical recording of whole-brain activity to reveal the neural mechanisms involved in prey hunting and capture. In this review we discuss how the larval zebrafish brain processes visual information to identify and locate prey, the neural circuits governing the generation of motor commands in response to prey, how hunting behavior can be modulated by internal states and experience, and some outstanding questions for the field.

## 1. Introduction

All animals must evaluate and interpret sensory inputs in order to respond with appropriate behaviors. The sensory environment is often complex and noisy, requiring sophisticated filtering to extract relevant information. Understanding how different stimulus attributes influence neural representations and thus sensory-motor processing is critical for understanding ecologically relevant decision making (Hein, 2022). A model system which offers several key advantages for studying these questions is the larval zebrafish. Zebrafish are vertebrates with strong anatomical and functional homology to the mammalian brain. They are amenable to genetic modification, allowing the development of transgenic lines which enable optical recording of neural activity in all or a chosen subset of neurons. Larval zebrafish can be engineered to be transparent, allowing unfettered optical access to whole-brain activity at single-neuron resolution *via* both 1-photon and 2-photon imaging of calcium or voltage indicators. Furthermore they also display a relatively rich behavioral repertoire from a very early age, including a capacity for selective attention (Patterson et al., 2013; Orger and de Polavieja, 2017; Bollmann, 2019; Fernandes et al., 2021).

One particularly interesting innate behavior displayed by larval zebrafish is hunting of single-celled organisms such as *Paramecia*. Hunting begins at around 5 days post-fertilization (dpf), and involves a series of rapid movements to intercept and ingest prey which can be moving rapidly (Box 1). This behavior relies predominantly on vision, with several brain regions playing key roles (Box 2). Hunting can be straightforwardly recorded at high temporal resolution (~500 fps) in 2D, and 3D recording is also possible (Bolton et al., 2019; Mearns et al., 2020). Unparalyzed, unaesthetized larvae can be immobilized in agarose for neural recording; up to around 14 dpf they breathe primarily through their skin and so no water flow across their gills is required. The tail can be freed from the agarose and behavior *via* tail movements recorded simultaneously with neural imaging. Assays of higher technical complexity allowing neural imaging during free-swimming behavior have also recently been introduced (Cong et al., 2017; Kim et al., 2017). Thus, hunting in larval zebrafish offers a unique opportunity to delineate the neural circuits involved in sensory-motor transformations at brain-wide scale.

**Figure 1:**
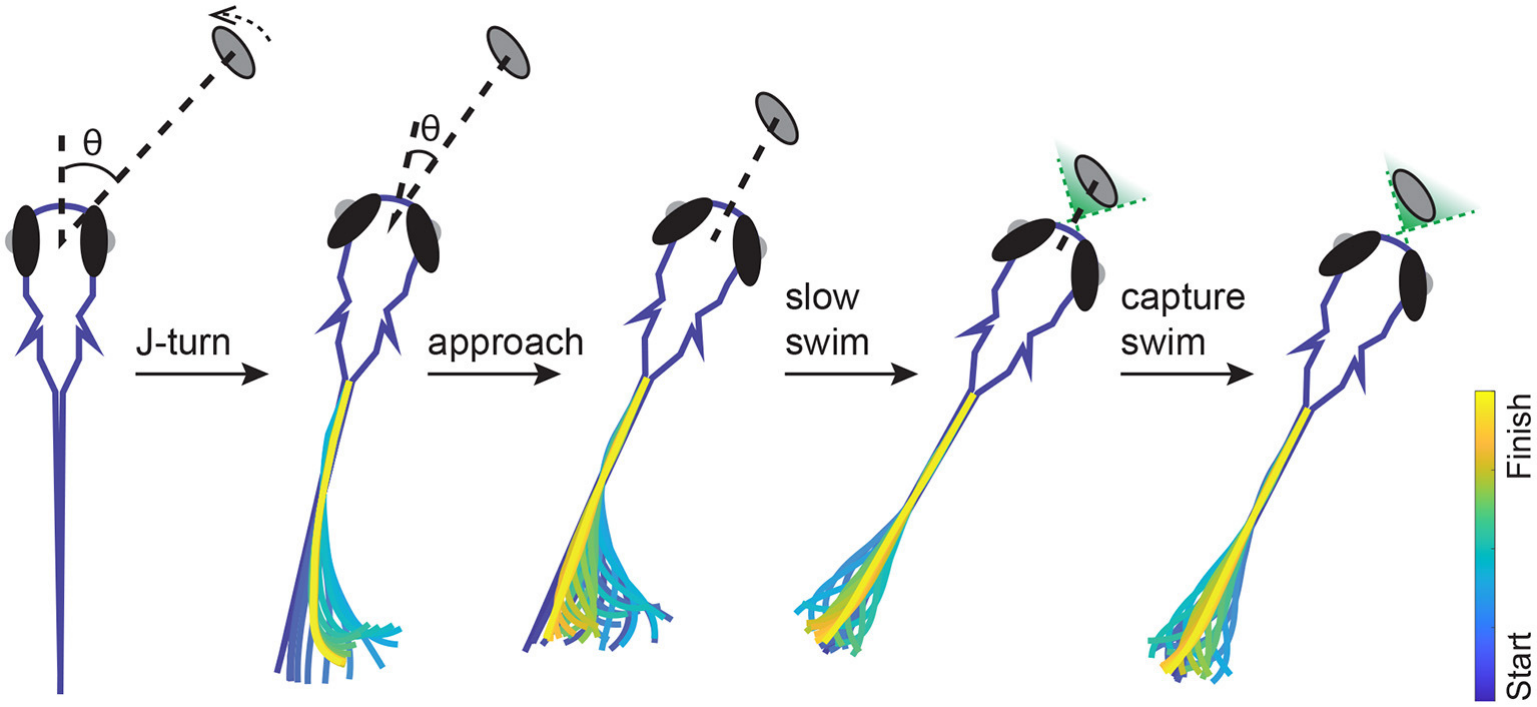
Typical example of the movement sequence during a hunting event. The fish detects a *paramecium*, then makes a J-turn toward the target. After the initial J-turn, the fish reduces the angle between itself and the target but the distance remains largely unchanged. The fish then adjusts its alignment with the target and reduces the distance using approach and slow swims. Once the target is within the strike zone (green shaded binocular region) the fish uses suction or a capture swim to try to capture the target.

Box 1 **Hunting behavior in larval zebrafish (Figure 1).**

Hunting behavior begins at 4–5 dpf, shortly after larval zebrafish start to swim. Larval swimming motions consist of brief (≈100–180 ms) bouts of activity followed by inactivity lasting up to 1–2 s. A typical hunting event takes about 1–3 s and consists of about 1–10 bouts; the magnitude and variability of these numbers decrease over development (Avitan et al., 2020). Larval swim bouts can be clustered into roughly 7–13 specific types (Marques et al., 2018; Mearns et al., 2020). These broad categories provide a useful summary of the behavioral space, and allow analyses of, for instance, the relative frequency of different bout types and transitions during hunting vs. exploratory behavior (Mearns et al., 2020). However, even bouts of the same type can show substantial variability from movement to movement. It is therefore likely that these different swimming patterns are drawn from a continuum of behavioral possibilities (Patterson et al., 2013; Trivedi and Bollmann, 2013; Mearns et al., 2020). Prey are most commonly detected at distances of ~ 3 mm and angles between 0 and 60° to the midline of the fish. A hunting sequence begins with eye convergence (Bianco et al., 2011; Muto and Kawakami, 2013), followed by a series of stereotypical swim bouts (Figure 1). Eye convergence serves to create a small binocular zone directly in front of the fish termed the strike zone (Gahtan et al., 2005; Bianco et al., 2011; Patterson et al., 2013). While this is clearly useful for the final bouts of the hunting sequence, why the eyes are converged for the initial bouts before the prey enters the strike zone is unclear (Avitan et al., 2020). The initial swim bouts following eye convergence act to align the larva's body toward the prey. These initial turning maneuvers are observed almost exclusively during hunting and are referred to as “J-turns” since the bending of the tail resembles the letter J (Borla et al., 2002; McElligott and O'Malley, 2005). Following these initial turns the larva uses a combination of J-turns, approach bouts and slow swim bouts to approach the prey, followed by either a rapid forward movement or suction to capture the prey (Borla et al., 2002; McElligott and O'Malley, 2005; Patterson et al., 2013; Mearns et al., 2020). During hunting, the bout pattern can be modeled as a marked renewal process. This reveals that previous bout patterns and prey locations influence behavioral choices on short time scales, and hunger state affects choices on longer time scales (Johnson et al., 2020). Larvae may also incorporate prediction into their behavioral strategies: models incorporating both prey position and velocity best capture real hunting trajectories, suggesting the fish could be estimating the future location of the prey (Bolton et al., 2019). However the turning angle and tail bending angle follow the kinematics of pure pursuit rather than parallel navigation (Soto and McHenry, 2020).

**Figure 2:**
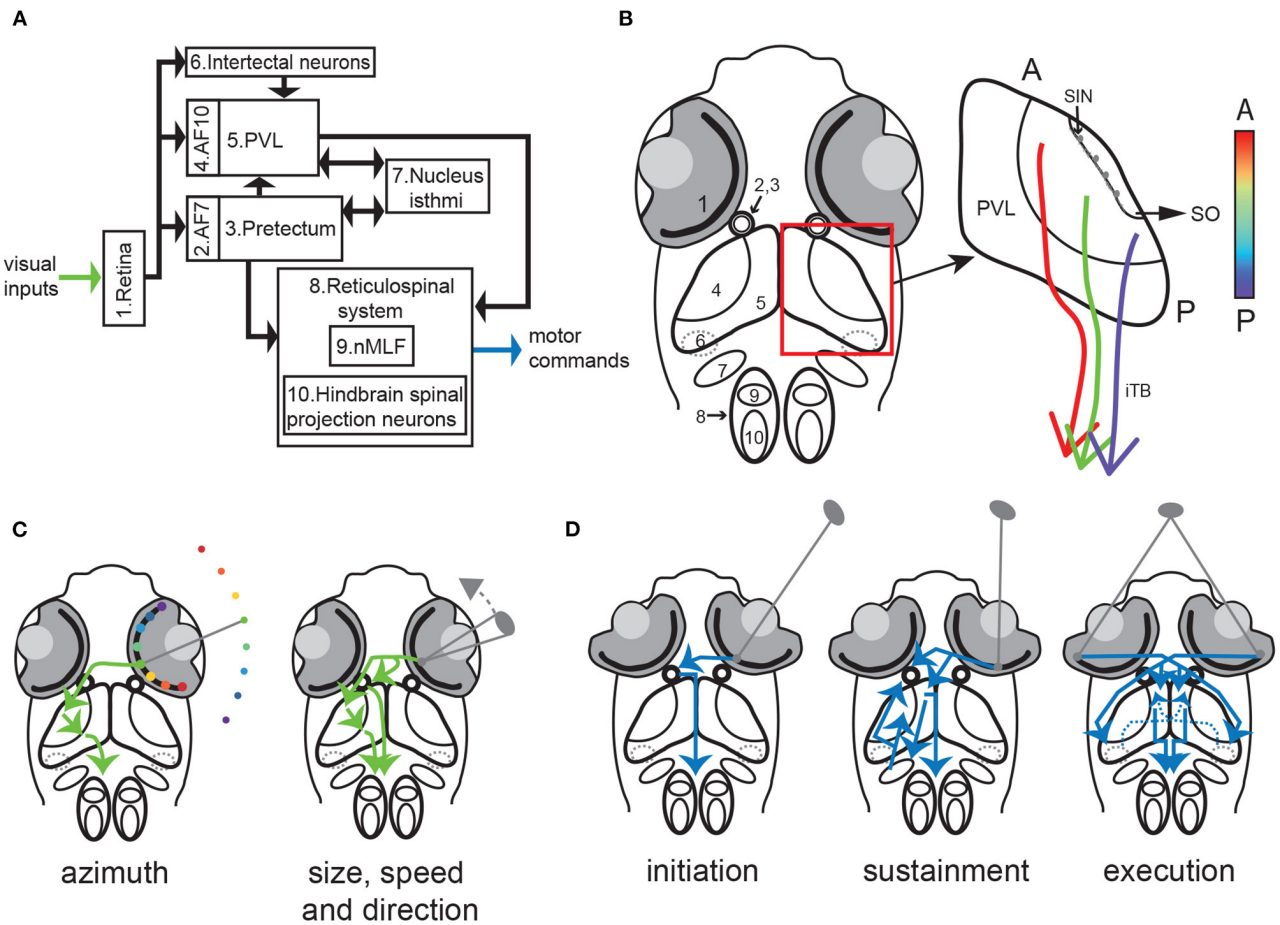
Circuits for hunting behavior. **(A)** schematic of the major components of the circuits underlying sensory processing and sensory-motor transformation during larval zebrafish hunting behavior. **(B)** Left: anatomical location of the major circuit components listed in a. Right: Schematic of the optic tectum (AF 10 and PVL). SINs are located in the SO layer of AF10. Projections to the hindbrain descend *via* the iTB, and the lateral iTB is topographically organized. The color bar represents topographic location along the anterior-posterior axis. A: anterior. P: posterior. **(C)** Schematic of circuit mechanisms underlying prey detection. Left: azimuth information is conveyed from retina to the optic tectum *via* AF10 first and then to the reticulorspinal system. Right: size and movement information are conveyed by selectively-tuned RGCs from the retina to AF7 and the optic tectum. **(D)** Schematic of circuit mechanisms underlying the generation of commands for hunting initiation (left), sustainment (middle) and capture (right). Left: Pretectal neurons send a command for initiating hunting to the reticulospinal system when prey target of appropriate size and speed is detected by the contralateral eye. Middle: Nucleus isthmi neurons receive input from and send feedback to the optic tectum and AF7 to potentiate neural activity for sustainment of hunting. Binocular detection of prey activates both the optic tectum and intertectal neurons to potentiate sensory signals for triggering execution of capture *via* the reticulospinal system. Dashed lines in **(B–D)** indicate that a structure/connection is ventral to the optic tectum.

Box 2 **Key circuit players in prey hunting in larval zebrafish (Figure 2).**

**Retina:** Similar to the mammalian retina (Wässle, 2004), the zebrafish retina contains 5 distinct cell types: photoreceptors, horizontal cells, bipolar cells, amacrine cells and retinal ganglion cells (RGCs). Photoreceptors consist of rods and cones, but unlike mammals zebrafish possess four cone types, with sensitivity to red, green, blue and UV light respectively (Chinen et al., 2003; Zimmermann et al., 2018). UV-sensitive cones are densely packed in the area temporalis (also known as the strike zone) giving rise to a fovea-like structure specialized for prey detection (Yoshimatsu et al., 2020). RGCs show selectivity for color contrast (Zhou et al., 2020), orientation (Nikolaou et al., 2012), speed (Semmelhack et al., 2014), direction (Gabriel et al., 2012) and size (Del Bene et al., 2010; Preuss et al., 2014; Semmelhack et al., 2014).

**Arborization Field 7 (AF7):** RGC axons terminate in 10 retinorecipient areas termed arborization fields (AFs) (Burrill and Easter, 1994; Baier and Wullimann, 2021). Of these the most important for hunting are AF10 (neuropil of the optic tectum, see below) and AF7, located ventral to AF10. RGC axons projecting to AF7 show selectivity to prey-like stimulus features (Semmelhack et al., 2014). Neurons residing in AF7 project to multiple downstream regions crucial to hunting behavior including the optic tectum, the nucleus isthmi, and the reticulospinal system. AF7 forms a specialized pathway to transmit information regarding prey detection and is crucial for the initiation of hunting behavior (Antinucci et al., 2019).

**Pretectum:** The pretectum is located in the caudal portion of the diencephalon, and contains several retinorecipient nuclei which are associated with some of the arborization fields (Yäñez et al., 2018; Baier and Wullimann, 2021). Pretectal neurons in the vicinity of AF7 are involved in the initiation of prey hunting (Antinucci et al., 2019).

**Optic tectum:** The optic tectum is the anamniotic equivalent of the superior colliculus, and since anamniotes lack a cortex constitutes the major sensory processing center of the brain (Nevin et al., 2010). The neuropil of the optic tectum (AF10) is a laminar structure in which most RGCs projections terminate, in layers specific to their functional properties (Xiao and Baier, 2007; Scott and Baier, 2009; Robles et al., 2014; Isa et al., 2021). Deep layers of the neuropil also receive inputs from regions including thalamus, hypothalamus, raphe nucleus, nucleus isthmi and cerebellum (Heap et al., 2013, 2018a,b; Filosa et al., 2016; Henriques et al., 2019; Fernandes et al., 2021). The neuropil contains a special group of inhibitory interneurons, the GABAergic superficial inhibitory neurons (SINs) which are located in a subdivision of the neuropil termed the stratum opticum. These neurons show selectivity to stimulus size, facilitating prey selection (Del Bene et al., 2010; Preuss et al., 2014). Most tectal neurons reside in the periventricular layer (PVL) where visually-responsive neurons are tuned for different parts of the visual field in a topographic manner (Niell and Smith, 2005; Kita et al., 2015; Romano et al., 2015; Avitan et al., 2020), and show mixed selectivity to prey-related visual features (Bianco and Engert, 2015; Thompson and Scott, 2016; Thompson et al., 2016; Helmbrecht et al., 2018). Tectal neurons project to multiple brain regions *via* the ipsilateral tectobulbar tract (iTB) (Henriques et al., 2019; Oldfield et al., 2020; Fernandes et al., 2021).

**Reticulospinal system:** The reticulospinal system contains about 150 spinal projections neurons in the midbrain and hindbrain (Lee and Eaton, 1991; Gahtan and O'Malley, 2003). It is the largest group of descending neurons involved in maintaining posture and generating swim movements (Kimura et al., 2013; Thiele et al., 2014; Orger, 2016). The cell bodies of most reticulospinal neurons reside within the hindbrain and are termed hindbrain spinal projection neurons. Reticulospinal neurons in the midbrain are located in the nucleus of the medial longitudinal fasciculus (nMLF) (Lee and Eaton, 1991). They receive inputs from visual areas including AF7 and the optic tectum, and project along the length of the spinal cord (Gahtan and O'Malley, 2003; Orger et al., 2008). nMLF neurons are involved in the generation of orienting swim bouts in zebrafish, especially during hunting (Gahtan et al., 2005; Thiele et al., 2014).

**Intertectal neurons:** Intertectal neurons are a group of GABAergic neurons with cell bodies located in the tegmentum, a multi-tissue structure in the midbrain positioned ventral to the optic tectum (Gebhardt et al., 2019). Intertectal neurons project to the deep layers of the neuropil in both hemispheres and respond to prey-like stimuli, with specific involvement in determining the location of the prey in the binocular strike zone.

**Nucleus isthmi:** The nucleus isthmi is a bilateral pair of cholingergic nuclei located in the tegmentum at the midbrain-hindbrain boundary. Neurons of the nucleus isthmi can be divided into two subtypes, one which projects ipsilaterally and one which projects bilaterally to AF7 and the optic tectum. Both subtypes receive ipsilateral inputs from optic tectum and AF7 (Wang, 2003; Henriques et al., 2019). The nucleus isthmi provides state-dependent feedback to sustain hunting and is involved in selective attention (Henriques et al., 2019; Fernandes et al., 2021).

## 2. Sensing what and where during hunting behavior

### 2.1. Hunting is driven by visual cues

Hunting behavior in larval zebrafish relies almost entirely on vision (Privat and Sumbre, 2020). In the dark hunting events are rare, and appear to be initiated by physical contact with the prey rather than visual cues (McElligott and O'Malley, 2005; Patterson et al., 2013). In immobilized preparations attempts to hunt are observed in response to visual presentation of prey-like stimuli (Bianco et al., 2011; Trivedi and Bollmann, 2013; Semmelhack et al., 2014, Bianco and Engert, 2015), and disruption of signaling in the visual pathway impairs the larva's ability to hunt (Gahtan et al., 2005; Del Bene et al., 2010; Semmelhack et al., 2014). The precise movements made by larvae during hunting are largely determined by prey location and movement; for instance initial turning speed and magnitude depend on prey location at detection (Patterson et al., 2013; Bolton et al., 2019; Avitan et al., 2020; Mearns et al., 2020), and subsequent bout selection is based on a tight stimulus-response loop (Mearns et al., 2020). Motor commands are carried downstream by the reticulospinal system, and are then translated into motor patterns *via* spinal cord circuitry (McLean et al., 2007; Bagnall and McLean, 2014; Berg et al., 2018).

### 2.2. Retinal specialization

Computation of relevant visual features begins in the retina. Photoreceptors have a spatial distribution that is adapted for the statistics of the zebrafish's natural environment (Zimmermann et al., 2018; Yoshimatsu et al., 2020), i.e., shallow rivers (Engeszer et al., 2007; Sundin et al., 2019). Chromatic information content is rich along the horizon and in the lower visual field, and the corresponding parts of the retina are densely packed with red, green and blue cones. The visual field above the fish is achromatic and the corresponding retinal region is dominated by rods. Detection of *Paramecia* under natural lighting conditions depends heavily on the UV cones in the retina (Novales Flamarique, 2013, 2016; Zimmermann et al., 2018; Yoshimatsu et al., 2020). The scattering of UV light by a *paramecium* makes it appear as a bright object on a dark background. UV cones are located predominately within a region facing slightly upward along the forward horizon which is specialized for prey detection, creating a fovea-like ‘strike zone' for prey hunting, and resulting in an achromatic representation of the visual world in this region (Zimmermann et al., 2018; Yoshimatsu et al., 2020). UV cones have elongated outer segments with slow recovery kinetics, anatomically and functionally resembling mammalian photoreceptors. They are also biased to on- (vs. off-) cells, enhancing their ability to detect UV-bright objects against a dark background. Similar spatial asymmetry is also observed for bipolar cells and retinal ganglion cells (RGCs), i.e., cells with UV-sensitivity are found mainly within the strike zone (Zimmermann et al., 2018; Zhou et al., 2020; Kölsch et al., 2021). The projection patterns of RGCs show functional specificity, as different types of RGCs have different arborization patterns to the optic tectum, pretectum, thalamus, and hypothalamus (Robles et al., 2014). Together, these spatial asymmetries and distinct projection patterns form the building blocks for selective visual-feature processing in downstream areas (Robles et al., 2013, 2014; Förster et al., 2020).

### 2.3. Predator or prey: Circuits for prey identification

To distinguish between predator and prey, zebrafish larvae primarily use information regarding the size and movement speed of the stimulus. Hunting behavior can be elicited using simple visual stimuli that mimic prey characteristics. Larvae tend to interpret small spots (5–8° in diameter) as prey (Del Bene et al., 2010; Semmelhack et al., 2014; Bianco and Engert, 2015) eliciting approach behaviors, and larger spots as predators eliciting avoidance behaviors (Bianco et al., 2011). Assessment of stimulus size begins with RGCs (Del Bene et al., 2010; Preuss et al., 2014; Semmelhack et al., 2014; Förster et al., 2020), and size-selective RGCs project specifically to the stratum opticum layer of the tectal neuropil. Superficial inhibitory neurons (SINs) in the stratum opticum consist of separate sub-populations with distinct size-tuning properties depending on their arborization pattern (Preuss et al., 2014). SINs also provide feedforward inhibition to tectal neurons and suppress tectal responses to full field stimuli (Del Bene et al., 2010). SINs that stratify in superfical neuropil are tuned to prey-like spots and provide lateral inhibition.

Neurons in the periventricular layer (PVL) of the optic tectum respond to a range of stimulus sizes from 3° to 30° (Niell and Smith, 2005; Förster et al., 2020). Their size tuning is partly inherited from RGCs, and ablation of RGCs tuned to specific sizes also abolishes PVL responses to these sizes (Förster et al., 2020). However, the size tuning of PVL neurons can be only partially predicted by a linear combination of RGC inputs, confirming that computation within the optic tectum refines the tuning properties of PVL neurons. A set of prey size-tuned RGCs also project to arborization field 7 (AF7) (Semmelhack et al., 2014), which in turn sends excitatory inputs to the stratum opticum. Ablation of these axons within AF7 disrupts hunting behavior. Therefore, the recognition of prey is likely computed synergistically between the optic tectum and AF7.

Prey movement is also an important cue for triggering hunting behavior. Larvae display eye convergence followed by approach when prey-sized spots are moving at 15-30°/s (Bianco et al., 2011; Bianco and Engert, 2015; Henriques et al., 2019). Prey size-tuned RGCs are also tuned for movement speeds similar to prey (Semmelhack et al., 2014), and this tuning is further refined in the optic tectum (Semmelhack et al., 2014; Förster et al., 2020). Tectal neurons show mixed selectivity to size and speed which is not a simple linear combination of the two features separately (Bianco and Engert, 2015). Specific assemblies of tectal neurons are active preceding or concurrent with oculomotor responses at the initiation of hunting response (Bianco and Engert, 2015). AF7 neurons also show preferential responses to size and speed similar to prey (Semmelhack et al., 2014).

### 2.4. Determining and tracking prey location

To determine the location of the prey at the initiation of a hunting event, the fish must estimate both the angle and distance of the prey. Azimuth location in the visual field projects topographically to the periventricular layer of the optic tectum (Niell and Smith, 2005; Kita et al., 2015). This topography is preserved in the projections of the lateral ipsilateral tectobulbar tract (iTB) from the tectum to the hindbrain (Helmbrecht et al., 2018), and activation of the iTB elicits swims toward the corresponding spatial location. However, elevation information is represented in a more complex manner, varying between different layers of the optic tectum and intermingled in the iTB. Azimuth information can be decoded more accurately from tectal activity by maximum likelihood decoding compared to a topographic decoder (Avitan et al., 2016), and the extent to which topographic vs. other decoding methods are normally used by zebrafish larvae remains unclear. How zebrafish larvae estimate prey distance beyond the small binocular zone (Bianco et al., 2011) is unclear, but could involve the perceived colorfulness of the target. In water chromatic contrast drops off with distance, providing a cue for how far away an object is (Wilkins et al., 2016). Asymmetries in tectal on- and off- response responses to light of different wavelength provide a potential neural mechanism for processing this chromatic contrast (Bartel et al., 2021).

After locating the prey the fish must keep track of its movement. Selectivity for the movement direction of visual stimuli begins in the retina, and the direction selectivity of tectal neurons is inherited from RGCs (Shi et al., 2017). RGCs with different direction selectivity terminate in different layers of the tectal neuropil, and the direction selectivity of PVL neurons is determined by the neuropil layer in which their dendrites arborize (Gebhardt et al., 2013; Lowe et al., 2013; Nikolaou and Meyer, 2015). Direction-selective PVL neurons receive inhibitory input corresponding to the non-preferred direction (Gabriel et al., 2012; Grama and Engert, 2012). RGCs show selectivity to upward, downward and caudal-to-rostral movement, while PVL neurons add an additional subgroup tuned to rostral-to-caudal movement (Nikolaou et al., 2012; Hunter et al., 2013). Computational modeling has demonstrated that this additional type of selectivity can be achieved by convergence of upward and downward excitatory inputs from RGCs and feedforward inhibition from SINs with selectivity to the non-preferred direction (Abbas et al., 2017; Yin et al., 2019).

Eye convergence causes a shift in the position of the prey image on the retina, which the fish must take into account. The velocity-to-position neural integrator (VPNI) could help keep track of both eye angle change during eye convergence (Miri et al., 2011; Lee et al., 2015; Brysch et al., 2019), and update and keep track of the spatial location of the prey (Helmbrecht et al., 2018). VPNI neurons are located in the hindbrain, and a subpopulation of these neurons in the abducens nucleus encodes primarily eye position information (Brysch et al., 2019). VPNI neurons project ipsilaterally and contralaterally to motor neurons and participate in coordinating tail movements (Lee et al., 2015). The iTB carrying information about prey location also projects in close proximity to VPNI neurons, suggesting involvement of the VPNI in tracking of spatial information (Helmbrecht et al., 2018). The torus longitudinalis may also help in tracking of prey location through maintenance of visual attention (Northmore, 2017). Torus longitudinalis neurons show two types of responses, one to change in luminance termed photic cells and one to saccades termed saccadic cells. Anatomically, torus longitudinalis is reciprocally connected with the optic tectum (DeMarco et al., 2020, 2021). A network model has been proposed that, *via* topographically localized excitation, the torus longitudinalis can help strengthen the responses in the tectal region that is predicted to be activated as a result of the saccade (Northmore, 2017). Although a functional validation of this model remains to be performed, it has been shown that feedback connections from torus longitudinalis to optic tectum play a critical role in binocular integration and spatial summation (Tesmer et al., 2022).

## 3. Sensory-motor decision making

Following prey detection and localization, the fish must then process this information to produce appropriate behavior. In order to make decisions during hunting the larva must accumulate sensory evidence (Shadlen and Newsome, 2001; Churchland et al., 2008; Groschner et al., 2018), but how this occurs in the zebrafish brain at timescales of <1 s is unknown. However recent work has revealed neural mechanisms of evidence accumulation over multi-second timescales during the optomotor response, when zebrafish change their orientation in response to full-field visual stimuli such as moving gratings. In particular whole-brain imaging has revealed clusters of neurons in the anterior hindbrain with roles corresponding to evidence integration, decision boundary thresholding and delivery of motor commands (Bahl and Engert, 2020; Dragomir et al., 2020). This suggests that zebrafish could be using a bounded integrator model (Ratcliff et al., 2016) in making sensory-motor decisions on this timescale.

### 3.1. Hunting initiation, sustainment and execution of capture

Motor commands to initiate hunting are generated in nucleus of the medial longitudinal fasciculus (nMLF) and hindbrain by a group of descending reticulospinal neurons (Gahtan et al., 2005; Orger et al., 2008). Bilateral ablation of two specific neurons in the nMLF, MeLr and MeLc, leads to impairment of hunting, as does unilateral ablation of the optic tectum and MeLr and MeLc on the opposite side. This suggests that these neurons receive inputs from the optic tectum on the contralateral side for initiating hunting (Gahtan et al., 2005). Unilateral activation of nMLF neurons elicits tail bending toward the stimulated side (Thiele et al., 2014). Therefore, nMLF is likely to be responsible for producing J-turns (Trivedi and Bollmann, 2013).

Two regions which might trigger these motor commands are the optic tectum and AF7, which both send descending connections carrying prey-related information to reticulospinal neurons (Semmelhack et al., 2014; Bollmann, 2019). While ablation of AF7 or optic tectum result in disrupted hunting behavior (Gahtan et al., 2005; Semmelhack et al., 2014), it is difficult to delineate the extent to which this is caused by an inability to generate motor commands vs. identify prey. Neurons involved exclusively in the execution of prey capture but not prey detection have been found in the pretectum (Antinucci et al., 2019), and ablation of pretectal neurons impairs hunting initiation (Muto et al., 2017). These neurons are only weakly activated by visual stimulation but display strong activity when hunting behavior is induced, and optogenetic activation of these neurons alone can initiate hunting.

Besides attempts to capture, hunting sequences can also be terminated by the fish appearing to give up and swim away, which have been termed abort events (Henriques et al., 2019; Avitan et al., 2020). The abort rate decreases over development as larvae become more experienced (Avitan et al., 2020). A crucial role in sustaining hunting is played by the nucleus isthmi (Henriques et al., 2019). In particular neurons in the nucleus isthmi are recruited by the initiation of hunting, and ablation of the nucleus isthmi results in a substantial increase in abort events. The nucleus isthmi is also involved in mediating stimulus competition for aversive stimuli (Schryver et al., 2020; Fernandes et al., 2021), but whether it plays a similar role during hunting behavior remains to be determined.

Execution of capture requires binocular integration, and the presentation of prey activates both contralateral and ipsilateral neuropil (Gebhardt et al., 2019). This activity co-localizes with the dendritic arbors of ITNs. These neurons form dendrites in the deep layers of the neuropil in both hemispheres and synapse onto inhibitory interneurons. Ablation of these neurons has no effect on hunting initiation but significantly increases the failure rate in executing capture (Gebhardt et al., 2019). These authors proposed that the execution of capture is only triggered upon co-activation of optic tectum in both hemispheres, together with the activation of these intertectal neurons.

### 3.2. Internal state-dependent modulation of predatory drive

Behavior is modulated by internal states (Flavell et al., 2022). Foraging behavior in zebrafish can be modulated by dynamic switches between exploration and exploitation states similar to those observed in humans under reinforcement learning strategies (Cohen et al., 2007; Dayan, 2012). Zebrafish larvae appear to spontaneously cycle between these states with a period of roughly 20 min, where the exploitation state is characterized by consecutive hunting sequences with a high success rate (Marques et al., 2020). This oscillation is independent of any obvious external stimulus. A group of serotonergic neurons in the dorsal raphe are activated shortly before each transition and appear to drive the transition. The activity of these neurons is negatively correlated with the activity of neurons related to the exploration state, such as a Gad1b cluster 13 in the hindbrain whose activity is related to routine turns, and positively correlated with neurons related to the exploitation state, such as a cluster of Vglut2 neurons in the cerebellum that drive eye convergence (Marques et al., 2020). The spontaneous oscillation can be interrupted by external stimuli such as luminance changes, and the exploitation state can be extended by successful capture of the prey. However, the purpose of this oscillation remains to be determined.

Hunger state can also affect hunting behavior. Starvation increases the upper limit of the size of objects regarded as prey, and also increases tectal responses to small objects (Filosa et al., 2016). Starved fish also spend a larger proportion of time with their eyes converged, and have increased eye divergence during exploration (Johnson et al., 2020). They also show reduced inter-bout intervals, increased usage of bouts associated with hunting such as J-turns, and increased transitions to high-effort exploratory bouts (Filosa et al., 2016; Muto et al., 2017; Wee et al., 2019). The change in sensitivity to object size is absent in a mutant line with impaired cortisol-mediated feedback to the hypothalamic-pituitary-interrenal axis (HPI axis, equivalent to the hypothalamic-pituitary-adrenal axis in mammals). As the HPI axis is responsible for regulating defensive behavior, interruption in feedback to this system could be the source of hunger-related behavioral changes. Starvation-related changes in tectal activity are linked to changes in activity in the HPI axis and in the serotonergic raphe neurons (Filosa et al., 2016). This is consistent with changes in neural activity in the hypothalamus, which contains the center of appetite control in zebrafish (Muto et al., 2017; Wee et al., 2019). The pretectum neurons that respond at detection of prey are connected with the hypothalamus, and interference with hypothalamic activity reduces feeding (Muto et al., 2017). The hypothalamus also contains circuits that regulate hunger and satiety (Wee et al., 2019). Together, these results suggest that hunger state modulates feeding behavior *via* alteration of visual responses in the tectum and such modulation is mediated by the HPI axis and the serotonergic system.

### 3.3. Experience-dependent plasticity in hunting behavior

Hunting behavior is also affected by experience during development. During normal development measures of hunting efficiency and success increase (Avitan et al., 2020), and dark rearing significantly disrupts hunting (Avitan et al., 2017). These improvements are also related to experience of hunting live prey (Lagogiannis et al., 2020; Oldfield et al., 2020). When compared with larvae that have not been fed or been fed only with dry food, larvae that have experience with live prey show improved hunting performance including increased hunting rate, reduced hunting event duration and increased success rate (Lagogiannis et al., 2020). Experienced larvae also utilize different hunting strategies. They show larger undershoot with their initial turn (turn angle to the prey is smaller than the prey azimuth) compared to non-experienced larvae. They also launch capture swims with faster swimming speeds and at larger distances to the prey (Lagogiannis et al., 2020). Experience of hunting live prey also increases the probability of hunting initiation compared to non-experienced larvae (Oldfield et al., 2020).

In terms of neural changes, experience alters the functional link between different brain regions with strengthened connectivity between pretectum and optic tectum, cerebellum, and hindbrain (Oldfield et al., 2020). A functional link between optic tectum and telencephelon is observed only in experienced larvae. Recruitment of telencephelon and habenula is observed during eye convergence upon visual stimulation of live prey only in experienced larvae. Furthermore, interrupting habenula activity in experienced larvae also reduces the probability of eye convergence upon prey stimulation (Oldfield et al., 2020). Together, these results suggest that the forebrain circuit is involved in mediating experience related improvements in hunting.

## 4. Conclusions and outstanding questions

To elucidate the neural mechanisms underpinning sensory-driven behavior requires linking neural representations across interconnected circuits with well-characterized motor outputs. Multiple animal models have been used to tackle these questions, each of which has different strengths and limitations. *Drosophila* have well-established circuit connectivity and have been used to study sensory-guided walking, flying and courtship behaviors (Seelig and Jayaraman, 2011; Calhoun and Murthy, 2017; Devineni and Scaplen, 2021). Dragonflies also have a relatively simple brain structure and have been used to study hunting behavior (Lancer et al., 2020). Rodents and non-human primates possess higher level cognitive abilities and have been used to study sensory-guided decision-making tasks (Churchland et al., 2008; Rauschecker and Scott, 2009; Freedman and Ibos, 2018). Zebrafish represent an intermediate point between insect and mammalian brains, and thus offer unique opportunities for studying neural control underlying behavior with a vertebrate brain structure with high homology to the mammalian brain. Hunting in larval zebrafish requires fine coordination between the visual and motor systems to ensure successful capture of the prey. Computation of prey-related visual features begins in the retina, followed by further refinement in downstream visual areas. Generation of subsequent motor behavior is linked to ongoing processing in visual areas. Multiple higher-order circuits participate in the modulation of hunting behavior. However many unanswered questions remain.

- How ecologically valid is our current understanding of larval zebrafish hunting behavior? The environments in which hunting has so far been studied in the lab are much simpler than the zebrafish's natural environment (Engeszer et al., 2007; Sundin et al., 2019), which generally includes a rich array of chromatic information, conspecifics, and multiple types of predators and prey.
- To what extent do the conclusions drawn about neural circuits from head-fixed preparations generalize to freely moving fish? Assays have begun to be introduced which allow neural imaging in moving fish (Cong et al., 2017; Kim et al., 2017; Marques et al., 2020). While these assays are a significant step forward, they currently require very shallow water (<1 mm depth) and rapid translation of the microscope stage to instantly cancel the movements of the fish so as to retain the brain within the field of view. There is a need for assays allowing brain imaging in fish hunting in a less constrained manner.
- How is information about prey position transmitted and transformed through the visual pathway to motor outputs? One hypothesis is that of topographic representations at each stage (Helmbrecht et al., 2018). However besides being suboptimal for decoding position (Avitan et al., 2016), the extent to which this principle holds through all stages of the sensory-motor transformation (for instance in the hindbrain) remains unclear.
- What causes hunting events to be aborted? Could it be due to distraction by other stimuli, an updated assessment of the probability of the likelihood of success, noise in sensory representation, or perhaps some varying combination of these?
- Why does the initial turn toward the prey tend to undershoot the target? This has been suggested to reflect a general hunting strategy fish employ for maximizing hunting efficiency (Bolton et al., 2019). However, the undershoot changes over development (Avitan et al., 2020) and is less prominent in fish with no prior experience of hunting (Lagogiannis et al., 2020), suggesting it is a learned strategy.
- How are competing stimuli prioritized? While this has begun to be investigated for aversive stimuli (Fernandes et al., 2021), how the fish selects between different appetitive stimuli is less clear.
- What is the purpose of the periodic oscillation between hunting and exploratory states, and how robust is this oscillation?
- How do neural representations vary between individuals, and how is this related to behavioral variations? A correlation has been demonstrated between hunting success and the quality of tectal representation of position (Avitan et al., 2020). However, an understanding of how individual variations in neural circuits lead to individual variations in hunting behavior is still very much in its infancy.
- What is the neural basis of evidence accumulation in natural prey hunting? While recent work has uncovered regions of the brain where evidence accumulation occurs in the context of the optomotor response (Bahl and Engert, 2020; Dragomir et al., 2020), the mechanisms underlying evidence accumulation during natural hunting remain unclear.
- How far in advance does neural activity predict behavior? Recent data from immobilized zebrafish has suggested that activity in the cerebellum can predict subsequent behavior many seconds in advance (Lin et al., 2020). However, whether this is also true of unconstrained hunting behavior is unknown. Since in free-swimming conditions zebrafish make bouts on a timescale of 1 per second, naively this would imply prediction of activity several bouts into the future, and there is currently no evidence for predictability of behavior on this timescale (Mearns et al., 2020).
- Most work in this area has focused on larvae because of the relative ease of brain imaging at these ages. However, behavioral patterns change as the fish develops (Westphal and O'Malley, 2013; Avitan et al., 2020), and much less is known about how the neural circuits driving hunting behavior mature as larvae become juveniles and then adults.

In summary, it is clear that zebrafish prey hunting still offers a rich seam of enquiry for understanding behavior, neural circuits, and most crucially the link between the two.

## Author contributions

All authors listed have made a substantial, direct, and intellectual contribution to the work and approved it for publication.
